# FGFR1 Expression and Role in Migration in Low and High Grade Pediatric Gliomas

**DOI:** 10.3389/fonc.2019.00103

**Published:** 2019-03-13

**Authors:** Naomi Egbivwie, Julia V. Cockle, Matthew Humphries, Azzam Ismail, Filomena Esteves, Claire Taylor, Katherine Karakoula, Ruth Morton, Tracy Warr, Susan C. Short, Anke Brüning-Richardson

**Affiliations:** ^1^Leeds School of Medicine, University of Leeds, Leeds, United Kingdom; ^2^Leeds Institute of Medical Research at St James's, University of Leeds, Leeds, United Kingdom; ^3^School of Medicine, Dentistry and Biomedical Sciences, Queen's University Belfast, Belfast, United Kingdom; ^4^Histopathology Department, Bexley Wing, St James's University Hospital, Leeds, United Kingdom; ^5^School of Biology, Chemistry and Forensic Science, University of Wolverhampton, Wolverhampton, United Kingdom

**Keywords:** pediatric gliomas, FGFR1, migration, immunohistochemistry, inhibitors

## Abstract

The heterogeneous and invasive nature of pediatric gliomas poses significant treatment challenges, highlighting the importance of identifying novel chemotherapeutic targets. Recently, recurrent Fibroblast growth factor receptor 1 (FGFR1) mutations in pediatric gliomas have been reported. Here, we explored the clinical relevance of FGFR1 expression, cell migration in low and high grade pediatric gliomas and the role of FGFR1 in cell migration/invasion as a potential chemotherapeutic target. A high density tissue microarray (TMA) was used to investigate associations between FGFR1 and activated phosphorylated FGFR1 (pFGFR1) expression and various clinicopathologic parameters. Expression of FGFR1 and pFGFR1 were measured by immunofluorescence and by immunohistochemistry (IHC) in 3D spheroids in five rare patient-derived pediatric low-grade glioma (pLGG) and two established high-grade glioma (pHGG) cell lines. Two-dimensional (2D) and three-dimensional (3D) migration assays were performed for migration and inhibitor studies with three FGFR1 inhibitors. High FGFR1 expression was associated with age, malignancy, tumor location and tumor grade among astrocytomas. Membranous pFGFR1 was associated with malignancy and tumor grade. All glioma cell lines exhibited varying levels of FGFR1 and pFGFR1 expression and migratory phenotypes. There were significant anti-migratory effects on the pHGG cell lines with inhibitor treatment and anti-migratory or pro-migratory responses to FGFR1 inhibition in the pLGGs. Our findings support further research to target FGFR1 signaling in pediatric gliomas.

## Introduction

Pediatric and adult gliomas are now recognized to be distinct disease entities. However, current standard of care for adult and pediatric high grade glioma (HGG) remains the same and both are associated with dismal disease outcome. Pediatric and adult gliomas are clearly distinct biologically, with differences in location, transformation rate [from low grade gliomas (LGG) to HGGs] and differences in tumor biology and genetic drivers (1). Pediatric glioma drug development must be based on a new understanding of the unique biology of these tumors, rather than simply employing adult drug strategies. Pediatric LGGs are strikingly characterized by oncogenic mutations in the *BRAF* gene leading to constitutive BRAF kinase activity (2). *In vivo* studies to target BRAF mediated signaling in other tumor types as well as first clinical trials in pediatric oncology have highlighted the importance of combination treatment targeting BRAF driven signaling (3, 4), one of such potential additional targets is the fibroblast growth factor receptor 1 (FGFR1). So far, there has been very little research into FGFRs in pediatric low and high grade gliomas. FGFRs comprise a group of membrane receptors involved in many cellular processes including proliferation and migration. High FGFR1 expression levels have been documented in many cancers including bladder and lung cancer due to gene amplification or deregulation at the transcriptional level (5, 6). In pediatric gliomas, genomic analyses have reported recurrent FGFR1 mutations (5, 6). Jones et al. sequenced blood and tumor tissues from pilocytic astrocytomas and identified FGFR1 mutations (7) with the mutational hotspots located on codons Asn546 and Lys656 (7, 8). Becker et al. reported that 6.7% of pilocytic tumors had FGFR1 point mutations on Lys656 and subsequently that tumors carrying the mutation had significantly poorer prognoses compared to wild-type variants (9). These studies support exploring FGFR1 as a potential genetic driver in pediatric glioma tumorigenesis (7, 8) and as a druggable target. All recent studies in pediatric glioma research have focused on FGFR1 at the genomic level with very little known about the role of FGFR at the protein level. Additionally, studies on FGFR1 and FGFR1 mutations have mainly concentrated on pediatric LGGs and further research is needed in pediatric HGGs (10, 11). This study aimed to firstly investigate FGFR1 and activated FGFR1 (pFGFR1) expression at the protein level in patient samples including pediatric and adult neurological malignancies where we identified an association of expression levels for FGFR1 and protein localization for pFGFR1 and malignancy. We screened patient derived and established pLGG and pHGG cell lines for the FGFR1 reported mutational hotspots and determined FGFR1 and pFGFR1 protein expression levels. We also studied the migratory/invasive behavior of low grade pediatric astrocytomas in comparison to HGGs since this is a prerequisite of disease progression. Finally, we assessed the role of FGFR1 protein expression and signaling in these processes with FGFR1 inhibitor studies. Our findings support a role for FGFR1 signaling in pediatric glioma migration with a potential for kinase signaling targeting: our TMA studies indicated an association of FGFR1 expression and malignancy and tumor grade; membranous pFGFR1 localization was also associated with malignancy and grade. Cell lines with high FGFR1 expression levels of both FGFR1 and activated FGFR1 possessed advanced migratory abilities. FGFR1 inhibitors had an anti-migratory effect on the two HGG cell lines whereas we observed an anti-migratory or potentially pro-migratory effect among the LGG cell lines. As all cell lines were FGFR1 wildtype we propose that FGFR1 amplification alone may contribute to disease progression.

## Materials and Methods

### Cell Lines

The established pediatric HGG cell lines SF188 (grade IV, GBM) and KNS42 (grade IV, GBM) were used. Five rare pediatric LGG patient-derived cell lines were studied including IN1591, IN2017, IN2356, IN2688, and IN1520. These cell lines were generated from grade I pilocytic astrocytomas obtained with informed consent during debulking surgery (REC 04/Q0508/98). The age of the patients ranged from 2 to 7 years at diagnosis. There were 1 female and 4 male patients. Tumor locations included temporal parietal lobe, frontoparietal lobe, posterior fossa, and the cerebellum. Patient-derived cell lines were immediately prepared in the laboratory from approximately 10 mg of excised biopsy, directly adjacent to tumor tissue processed for routine histological evaluation tissue as previously described (12). Cells were used for experiments between passages 1–4.

All cell lines were verified by in-house short tandem repeat (STR) profiling service and were mycoplasma free.

### Cell Culture

KNS42 and SF188 were grown in complete Dulbecco's Modified Eagle's Medium (DMEM) (Sigma) supplemented with 10% heat-inactivated fetal calf serum (HI-FCS) (Labtech) as previously described (13). IN1591, IN2017, IN2356, IN2688, and IN1520 were grown in Ham's F-10 Nutrient mixture (1X) (Gibco) supplemented only with 10% HI-FCS (Labtech). F-10 medium was the preferred choice for the patient derived samples as the glucose concentration was more comparable to *in vivo* glucose levels but medium composition did not significantly differ otherwise. Global expression profiling had demonstrated that short-term cultures derived from pediatric pilocytic astrocytoma are representative of the tumors *in situ* when grown with serum (14). The cells were passaged at 1:2 to 1:3 dilutions weekly or bimonthly.

### Inhibitors

The inhibitors used were Ponatinib (Selleckchem), SSR128129E (Selleckchem), and BGJ398 (Selleckchem). All inhibitors were diluted in dimethylsulphoxide (DMSO) (Sigma).

### Screening for FGFR1 and Activated FGFR1 (pFGFR1) Expression Levels in the Tissue Microarray (TMA)

A commercially available tissue microarray (TMA) (CNS2081, US Biomax) with 208 cores obtained from CNS pathologies was screened for FGFR1 and pFGFR1 expression by immunohistochemistry (IHC). The TMA included adult and pediatric HGGs, LGGs, meningiomas, and medulloblastomas alongside healthy controls. Prior to staining, the TMA slide was heated at 70°C before immersion in xylene. Rehydration in absolute ethanol (Sigma) was followed by 90% ethanol and finally 70% ethanol. Enzyme antigen retrieval was achieved using Bond Enzyme Pre-treatment Kit (Leica Biosystems). Endogenous peroxidase activity was blocked using Bloxall (Vector Laboratories). Protein blocking was then performed with 10x casein (1:10) (Vector Laboratories). Rabbit anti-FGFR1 primary antibody (Sigma) or anti pFGFR1 (Cell signaling) was added at 1:3,000 and 1:100 concentrations followed by incubation with the anti-rabbit polymer horseradish peroxidase (HRP) secondary antibody (MenaPath). The slide was developed with ImmPACT 3,3'-diaminobenzidine (DAB) HRP substrate (Vector Laboratories) and counterstained using in-house Mayer's haematoxylin, dehydrated and mounted with DPX. An Aperio scanner (Leica Microsystems, Milton Keynes, UK) was used for visualization and scoring of the stained cores.

### Preparation of 3D Spheroids for Immunohistochemistry

Spheroids embedded in collagen were prepared after completion of migration/invasion experiments according to Cheng et al. (15). Sections of spheroids were stained for the expression of Ki67 (proliferation marker) (ThermoScientific), Cleaved caspase 3 (apoptosis marker) (Cell signaling), FGFR1, and pFGFR1 (Sigma, Cell Signaling).

### Immunohistochemistry Scoring

The TMA slides were digitally scanned using in-house virtual pathology services. Immunostaining was quantified using manual and computerized techniques. The immunostaining of each core was scored by eye using two scoring methods; intensity of staining: 0—no staining, 1—weak staining, 2—medium staining and 3—strong staining, and counting the number of cells stained for a marker and expressing as a proportion: 0–0, 1–25%−1, 26–50%−2, 51–75%−3, 76–100%−4. An overall score, out of 7, was calculated combining intensity of the immunostaining and percentage of the core stained. Scores were dichotomized into low and high FGFR1 expression using STATA 13.1 (StatCorp). For automated scoring, Aperio ImageScope (Leica Biosystems) was used to generate positive pixel count image analysis of each TMA core. Pre-set 1,400 × 1,400 (width by height) boxes were used. Each core was analyzed individually and generated a “positivity” value, which quantified the percentage of immunopositive staining present. The positive pixel count percentage was given an overall score, out of 7, using: Positive Pixel Count Percentage % Score 0–12.5 = 0; 12.6–25 = 1; 26–37.5 = 2; 37.6–50 = 3; 51–62.5 = 4; 62.6–75 = 5; 76–87.5 = 6; 87.6–100 = 7.

STATA 13.1 was used to dichotomize the scores into low and high FGFR1 expression. The sections containing the spheroids and migratory cells encased in collagen were analyzed and confirmed by the pathologist (Azzam Ismail) and the individual cells within the spheroids identified by their haematoxylin stain. Identified individual cells within the spheroids were counted manually and recorded as being stained or not stained with the individual marker for protein expression. All antibody concentrations had been pre- optimized using negative and positive control tissues. Scoring of the spheroid sections was achieved by counting the number of cells with protein expression and expressing as % of cells stained, also noting cellular localization of the marker within the spheroids, for example nuclear, cytoplasmic and membrane associated. Cleaved caspase 3 and Ki67 were recorded as a proportion of cells scored and then divided into high (>50% stained) and low (<50% stained) expression.

### Q-PCR Detection of BRAF Fusion

Total RNA was extracted from five patient-derived cell cultures of pLGG using an RNeasy Mini kit (Qiagen Ltd., Manchester, UK) and reverse transcribed to cDNA using a QuantiTect Reverse Transcription Kit (Qiagen) according to the manufacturer's instructions. Q-PCR was performed using 2.5 μl of cDNA from each culture, primers and TaqMan probes specific for the KIAA1549-BRAF fusions 16–9, 15–9, and 16–11 (Assays-on-Demand Gene Expression products Hs04396516_ft, Hs04421337_ft and Hs04396507_ft) and TaqMan Gene Expression Mastermix on an ABI 7500 HT Sequence Detection System (Applied Biosystems, Warrington, UK). Glyceraldehyde-3- phosphate dehydrogenase (*GAPDH*) was used as an endogenous control (Assays-on-Demand Gene Expression product Hs02758991). Amplification conditions were 2 min at 50°C, 10 min at 95°C, and then 50 cycles of 15 s at 95°C and 1 min at 60°C. Fluorescence was recorded and cycles to threshold (C_T_) were calculated using 7500 Sequence Detection Software version 2.3 (Applied Biosystems). Reactions were run in triplicate in singleplex reactions.

### DNA Extraction for FGFR1 Mutation Screening

DNA was extracted from cells using the Qiagen DNeasy kit according to manufacturer's instructions.

### Mutation Screening of FGFR1 Mutation Hotspots

PCR primers were designed using Primer3 (http://bioinfo.ut.ee/primer3-0.4.0/). Primer set F1 targeted codon 546; primer set F2 targeted codons 655–658 of reference cDNA sequence NM_023110 with ATG_i_ as codon 1. All primers had binding sites in the introns flanking the exons containing the target codons. Primers sequences were: F1F tcaagtcccagggaaaagca; F1R gggcagggaaagccagtct; F2F gagccttccagctccctcac; F2R ccaccccactccttgcttct. PCR was carried out using HotStarTaq master mix (Qiagen) in 10 μl final volume using 40 pmol of each primer, 10% DMSO and 10 ng of DNA. Reaction conditions were 95°C for 15 min, 36 cycles of [95°C, 30 s; 58°C 30 s; 72°C, 30 s], followed by 72°C for 10 min and hold at 15°C. All samples were bi-directionally sequenced using Big- Dye terminator chemistry version 1.1 (Applied Biosystems). PCR primers were used for sequencing. Data collection was performed using an Applied Biosystems 3130xl Genetic Analyser. Data analysis was carried out by visual inspection of electropherograms and using Mutation Surveyor 3.2 (SoftGenetics).

### Immunofluorescence of the Cell Lines

Immunofluorescence was used to determine FGFR1 and pFGFR1 expression in 2D monolayers of all cell lines. Cells were fixed with 4% paraformaldehyde and permeabilized with 0.1% PBS-Triton-X-100 (Sigma). After blocking with 0.1% Marvel skimmed milk powder, the cells were incubated with the primary antibody, rabbit anti-FGFR1 (1/100, Cell Signaling) or mouse anti-pFGFR1 primary antibodies (1/500, Novus Biologicals) in blocking solution. The secondary antibodies, Alexa Fluor 488 anti-rabbit or anti-mouse (1/500, Molecular Probes), were added at 4 mg/ml alongside Alexa Fluor 594 Phalloidin stain (Molecular Probes) in blocking solution.

### Image Quantification and Analysis

Immunofluorescence images of the cells were generated with the EVOS imaging system (Advanced Microscopy Group) set at the same magnification (x40) to allow cell identification and comparison across the cell lines analyzed. To guide fluorescence quantification the cells were incubated with the specific antibody (anti-FGFR1, pFGFR1) and co-stained with the nuclear marker DAPI (Molecular Probes) and Alexa Fluor phalloidin 594 (actin) (1/500, Molecular Probes) to highlight the overall cell morphology for identification of individual cells. For image acquisition for each specific marker (FGFR1, pFGFR1) imaging settings (aperture, exposure time) were obtained to allow fluorescence capturing at optimal exposure, avoiding under or over-exposure. The same settings were then applied to all cells to be analyzed within a group, for example, for all cell lines unstimulated vs. stimulated stained for FGFR1, on the same day for normalization and to allow comparison of protein levels. At least 50 cells/antibody/ three repeats were analyzed; for the LGG cell lines we scored all the cells within a slide due to low proliferation and for the HGG cell lines at least 200 cells from 4 randomly selected fields within the slide. Fluorescence images of the cells were captured as .tif images and analyzed using ImageJ fluorescence quantification. For this, cells were identified by their actin and DAPI stains and selected for measurements with the drawing tool. The mean gray value and integrated density were measured. Background readings within the individual slides were also taken. The corrected total cell fluorescence (CTCF) was then calculated by using the following formula: CTCF = Integrated Density – (Area of selected cell x Mean fluorescence of back ground readings).

### Two-Dimensional Random Cell Migration

For live cell imaging of random cell migration cells were seeded at 1 × 10^5^/well in flat-bottomed 96 well plates (Nunc) with or without inhibitor treatment in full medium, serum-free Heparan sulphate proteoglycan (HSPG) (Sigma) only supplemented medium or serum-free HSPG/FGF2 supplemented medium. FGF2 (Sigma) was added at 10 ng/ml and HSPG (Sigma) at 10 μg/ml. Cell migration was imaged with the IncuCyte ZOOM system (Essen BioScience) over 72 h at hourly intervals at x10 magnification. Avi formatted movies were analyzed using ImageJ^1^.

For SF188 and KNS42, at least 20 cells per condition/recorded field were identified and random migratory patterns recorded by tracking individual nuclei with MTrackJ (https://imagescience.org/meijering/software/mtrackj/) to determine velocity and directionality (16). For pLGG, up to 10 cells/condition/field were identified because of low cell proliferation rates. Rose plots were generated using Ibidi Chemotaxis PlugIn.

### Three-Dimensional Spheroid Migration Assay

Cells were seeded at 5 × 10^3^ cells per well in low adherence round bottomed 96 well plates (Nunc) in full media, serum-free HSPG (Sigma) only supplemented media or serum free HSPG/FGF2 supplemented media. After spheroid formation, the supernatant was replaced with a mixture of collagen (Sigma), DMEM (Sigma) growth medium for the high-grade cell lines or Ham's F-10 (1X) (Gibco) for the low-grade cells. 1M sodium hydroxide (NaOH) (Sigma) was added to initiate collagen polymerization. Each well was treated with a predetermined inhibitor concentration or DMSO (Sigma) in at least three repeats and separate experiments. Images were taken at 0, 24, 48, and 72 h to monitor spheroid invasion and migration using the EVOS Cell Imaging System (Advanced Microscopy Group) at 4x magnification. ImageJ was used to analyse the images. An overall migration index (MI) was calculated, as previously described by Cockle et al. (13).

### Statistical Analysis

Independent unpaired *t*-tests were used to analyse the data from the cell migration and 3D invasion assays. Immunofluorescence was analyzed using Image J. Microsoft Excel was used to calculate CTCF to determine the FGFR1 and pFGFR1 expression in the cell lines. For the TMA generated data, a Pearson's chi squared test was conducted to investigate the association between high or low FGFR1 expression with tumor grade, location, tumor type, gender, and age using GraphPad Prism. Both the dichotomized manual and computerized IHC scores were used. *P* < 0.05 was considered statistically significant. For analysis of the pFGFR1 localization TMA data Fisher's exact test was used.

## Results

### FGFR1 Expression and pFRGR1 Localization Is Associated With Tumor Grade and Malignancy

**Figure 1:**
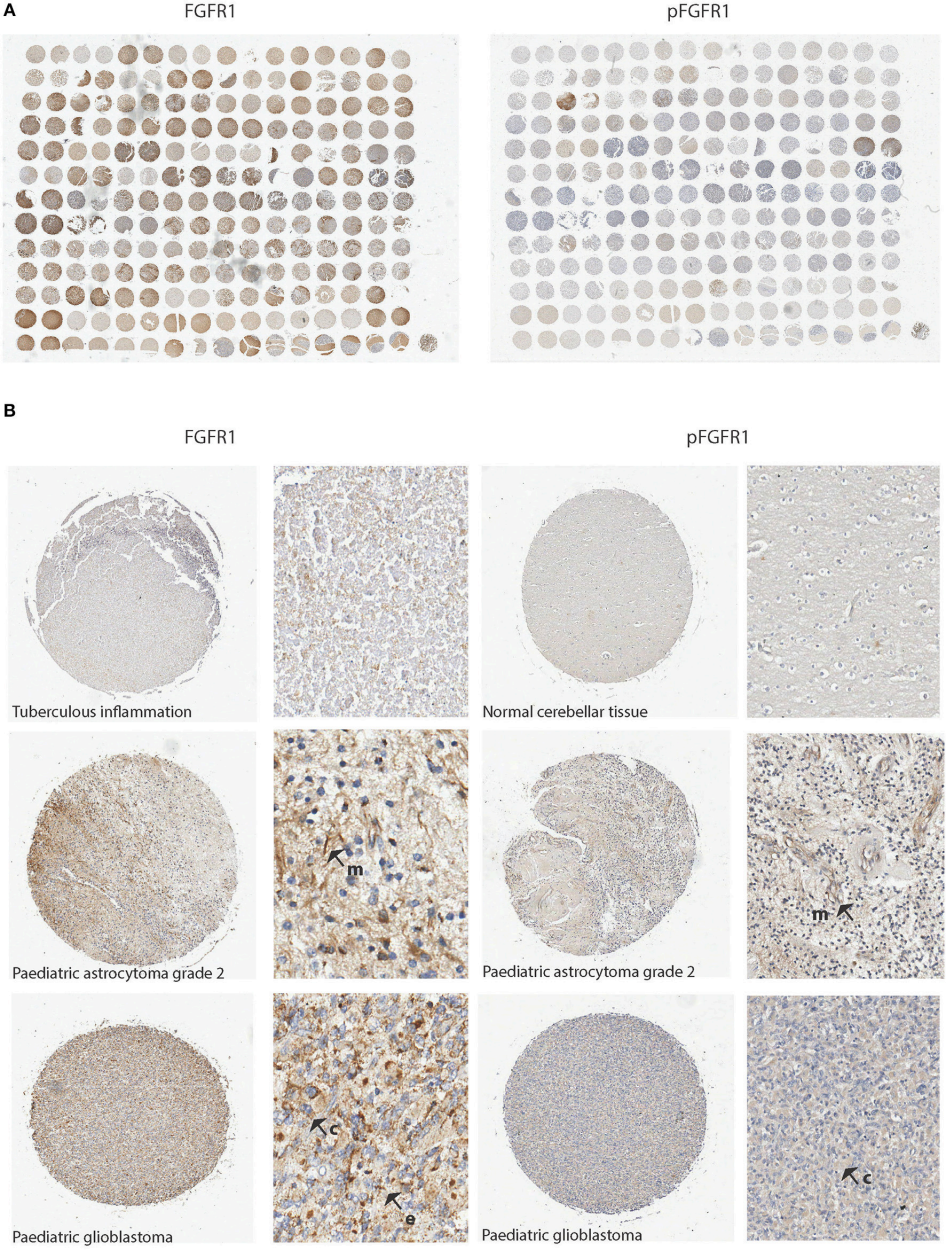
FGFR1 levels vary in tumor tissue samples from patients as determined by IHC. **(A)** Scanned high resolution image of the TMA stained for FGFR1 and pFGFR1. Different intensities of antibody labeling were observed as indicated by brown staining. **(B)** Examples of strong and weak staining in TMA cores at low and high magnification. Membranous and cytoplasmic labeling was observed. Top core shows a representative sample for negative stain (tuberculous inflammation) or normal cerebellar tissue; the middle core represents a sample from a pediatric astrocytoma grade 2 and the bottom an example of a high grade pediatric glioma (Individual cores × 10; magnified examples × 40). Arrows indicate staining patterns. m = membranous; c = cytoplasmic; e = extracellular.

The purpose of the TMA analysis here was to confirm FGFR1 expression at the protein level and to validate clinical relevance of FGFR1 expression and to investigate pFGFR1 expression levels. Both adult and pediatric gliomas were included in the TMA. Eight TMA cores were excluded because there was insufficient tissue present to analyse. Using manual and computerized scores there were clear differences in staining intensity and percentage of cells stained indicating varying expression levels for FGFR1 and localization for pFGFR1 (Figure 1A). Apart from membranous labeling cytoplasmic and extracellular FGFR1 staining was also observed (Figure 1B). The ages were divided into childhood and young adult (i.e., up to 25 years old), adult (26–60 years old), and elderly populations (over 60 years old). There was significantly low FGFR1 expression in childhood and young adulthood tumors than the other age groups (*p* = 0.04). There was a significant correlation between high FGFR1 expression in the cerebrum compared to the cerebellum. Patient samples obtained from the cerebrum had higher FGFR1 expression (*p* < 0.0001) than the cerebellum as determined by manual scoring and digitized scoring (*p* = 0.02) (Table 1). There was a significant positive association between malignancy and FGFR1 expression. Malignant tumors had significantly high FGFR1 expression compared to benign tumors by digital analysis (*p* < 0.001; Table 1). There was also significantly high FGFR1 expression in grade 2 tumors (*p* = 0.02) indicative of a role in tumorigenesis and disease progression (Table 2). Table 3 summarizes data obtained for the normal controls. We observed two distinct protein localizations of pFGFR1, cytoplasmic and membranous; there was a significant over-representation of membranous staining in the malignant cases (38%) compared to the benign (9%). Additionally, for the negative cases there was an overrepresentation of no staining in the benign (68%) compared the malignant (42%) (*p* = 0.024). There was an over-representation of negative staining in grade one compared with higher grades. Additionally, membranous staining was significantly associated with higher grade cases (*p* = 0.010) (Tables 4, 5).

**Table 1:** TMA results for FGFR1 expression reveal associations of FGFR1 expression levels with age, tumor location and malignancy.

	**Manual analysis FGFR1**			**Digital analysis**		
**Clinical characteristic**	**Low FGFR1 expression (*n* = 46)**	**High FGFR1 expression (*n* = 50)**	***p*-value**	**Low FGFR1 expression (*n* = 53)**	**High FGFR1 expression (*n* = 43)**	***p*-value**
**Age**			*p* = 0.04			*p* = 0.13
25 and under	12 (26.1%)	4 (8.0%)		12 (22.6%)	4 (9.3%)	
26–60	29 (63.0%)	42 (84.0%)		35 (66.0%)	36 (83.7%)	
Over 60	5 (10.9%)	4 (8.0%)		6 (11.4%)	3 (7.0%)	
**Sex**			*p* = 0.99			*p* = 0.51
Male	24 (52.2%)	26 (52.0%)		26 (49.1%)	24 (55.8%)	
Female	22 (47.8%)	24 (48.0%)		27 (50.9%)	19 (44.2%)	
**Location**			*P* < 0.0001			*p* = 0.02
Cerebrum	33 (71.7%)	50 (100.0%)		42 (79.2%)	41 (95.3%)	
Cerebellum	13 (28.3%)	0 (0.0%)		11 (20.8%)	2 (4.7%)	
**Tumor type**			*p* = 0.89			*p* < 0.001
Benign	11 (23.9%)	11 (22.0%)		19 (35.8%)	3 (7.0%)	
Malignant	29 (63.0%)	31 (62.0%)		27 (50.9%)	33 (76.7%)	
Unknown	6 (13.0%)	8 (16.0%)		7 (13.2%)	7 (16.3%)	

**Table 2:** FGFR1 expression is associated with tumor grade in astrocytomas, with high expression found in grade 2 tumors.

	**Manual analysis FGFR1 astrocytomas only**			**Digital analysis**		
**Clinical characteristic**	**Low FGFR1 expression (*n* = 12)**	**High FGFR1 expression (*n* = 20)**	***p*-value**	**Low FGFR1 expression (*n* = 8)**	**High FGFR1 expression (*n* = 24)**	***p*-value**
**Age**			*p* = 0.77			*p* = 0.46
25 and under	2 (16.7%)	2 (10.0%)		1 (12.5%)	3 (12.5%)	
26–60	8 (66.7%)	16 (80.0%)		5 (62.5%)	19 (79.2%)	
Over 60	2 (16.7%)	2 (10.0%)		2 (25.0%)	2 (8.3%)	
**Sex**			*p* = 0.26			*p* = 0.40
Male	6 (50.0%)	14 (70.0%)		6 (75.0%)	14 (58.3%)	
Female	6 (50.0%)	6 (30.0%)		2 (25.0%)	10 (41.7%)	
**Location**			*P* = 0.06			*p* = 0.40
Cerebrum	10 (83.3%)	20 (100.0%)		8 (100.0%)	22 (91.7%)	
Cerebellum	2 (16.7%)	0 (0.0%)		0 (0.0%)	2 (8.3%)	
**Tumor grade**			*p* = 0.90			*P* = 0.02
1	2 (16.7%)	2 (10.0%)		2 (25.0%)	2 (8.3%)	
2	4 (33.3%)	8 (40.0%)		1 (12.5%)	11 (45.8%)	
3	2 (16.7%)	2 (10.0%)		0 (0.0%)	4 (16.7%)	
4	3 (25%)	5 (25.0%)		5 (62.5%)	3 (12.5%)	
Unknown	1 (8.3%)	3 (15.0%)		0 (0.0%)	4 (16.7%)	

**Table 3:** Data analysis of FGFR1 expression in normal controls does not reveal an association of FGFR1 expression.

	**Manual analysis FGFR1-controls**			**Digital analysis**		
**Clinical Characteristic**	**Low FGFR1 expression (*n* = 2)**	**High FGFR1 expression (*n* = 6)**	***p*-value**	**Low FGFR1 expression (*n* = 3)**	**High FGFR1 expression (*n* = 5)**	***p*-value**
**AGE**						
25 and under	0 (0.0%)	3 (50.0%)	N/A	0 (0.0%)	3 (60.0%)	*P* = N/A
26–60	2 (100.0%)	3 (50.0%)		3 (100.0%)	2 (40.0%)	
Over 60	0 (0.0%)	0 (0.0%)		0 (0.0%)	0 (0.0%)	
**SEX**						
Male	0 (0.0%)	1 (16.7%)	*p* = 0.54	0 (0.0%)	1 (20.0%)	*P* = 0.41
Female	2 (100.0%)	5 (83.3%)		3 (100.0%)	4 (80.0%)	
**LOCATION**						
Cerebrum	1 (50.0%)	2 (33.3%)	*P* = 0.67	2 (66.7%)	1 (20.0%)	*P* = 0.19
Cerebellum	1 (50.0%)	4 (66.7%)		1 (33.3%)	4 (80.0%)	

*Subcellular pFGFR1 expression association*.

**Table 4:** Data analysis of subcellular pFGFR1 expression reveals association with malignancy.

	**Malignant**	**Benign**	***p*-value**
Membranous	23 (38%)	2 (9%)	0.024
Cytoplasmic	12 (20%)	5 (23%)	
Negative	25 (42%)	15 (68%)	

*pFGFR1 expression associated with grade*.

**Table 5:** Data analysis of subcellular pFGFR1 expression reveals association with grade.

	**Grade 1**	**Grade 2**	**Grade 3**	***p*-value**
Negative	6 (100%)	3 (25%)	1 (20%)	0.010
Cytoplasmic	0 (0%)	3 (25%)	0 (0%)	
Membranous	0 (0%)	6 (50%)	4 (80%)	

### Low Grade Pediatric Gliomas Predominantly Express BRAF Fusion but Are FGFR1 Wild Type

The BRAF fusion was present in 4 of 5 (80%) patient-derived cell cultures of pLGG (IN1520, IN1591, IN2017, and IN2688), all of which expressed the KIAA1549-BRAF 15-9 fusion supporting a true representation of high BRAF frequency common to pLGGs in our *in vitro* models. The median C_T_ value for the 15-9 fusion in these 4 samples was 36.71 and 17.56 for GAPDH. The remaining pLGG sample, IN2356, did not express any of the 3 KIAA1549-BRAF fusions. None of the cell lines had FGFR1 mutations in the investigated mutational hotspots.

### Low Grade and High Grade Pediatric Gliomas Exhibit Different Migratory Phenotypes in 2D and 3D

**Figure 2:**
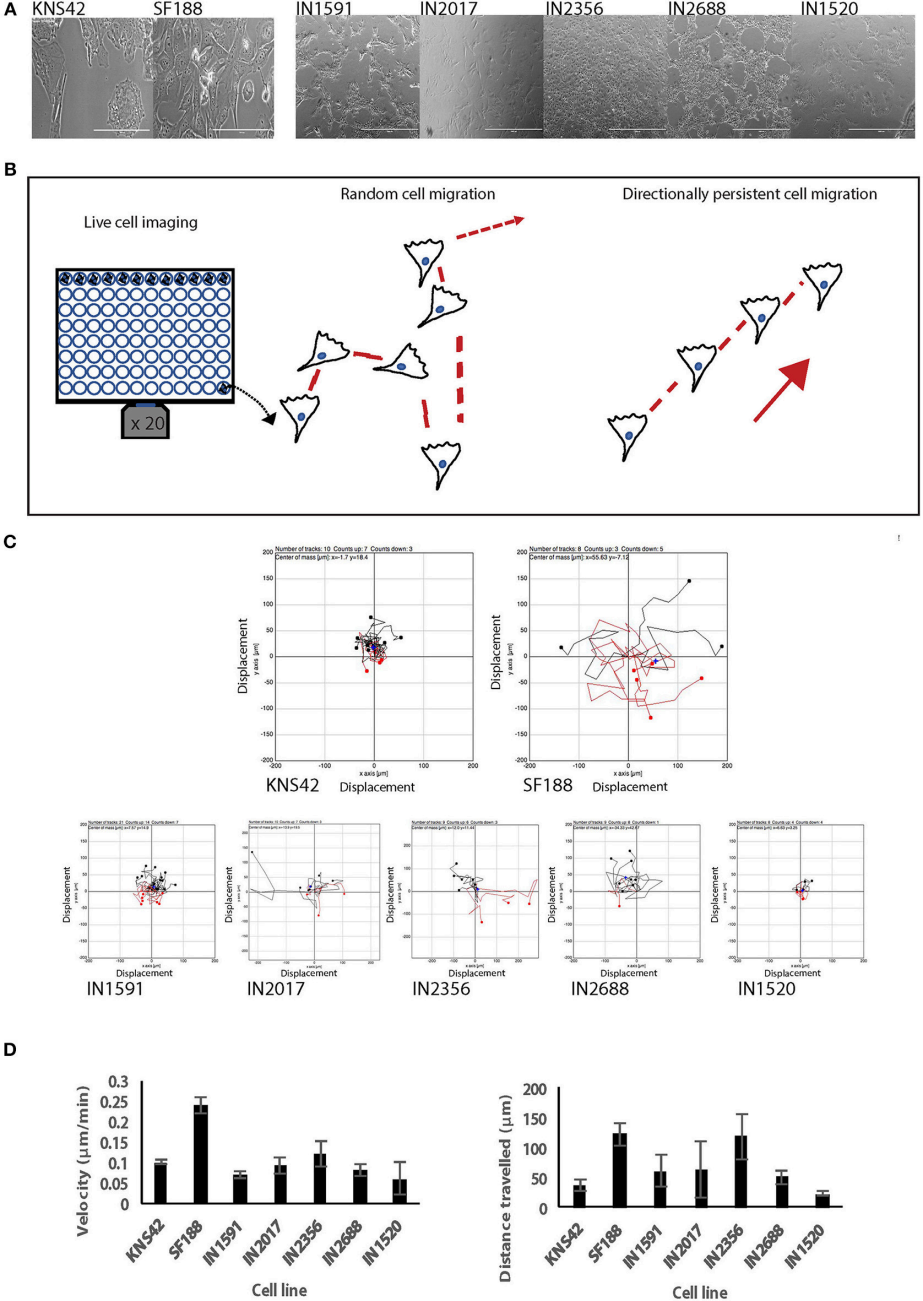
Low and pHGGs are able to migrate in a 2D environment as assessed by random cell migration. **(A)** Five pLGGs and 2 pHGG cell lines were used in this study. All cell lines were adapted to tissue culture and exhibited distinct morphologies. Scale bar = 1000 microns. SF188 and KNS42 magnification x 40. **(B)** Diagrams to illustrate random migration and directionally persistent migration. According to Petrie et al. (17), directionality is defined as the displacement divided by the total path length of the cell. If a cell is migrating more randomly, directionality decreases and vice versa. **(C)** Rose plots graphs generated for each cell line from individual cells after imaging of random cell migration observed over 72 h highlighting migratory ability among the cell lines. **(D)** Graphic representation of random cell migration of 2 HGGs and the five pLGGs as measured by velocity and distance traveled.

We firstly wanted to ascertain if a panel of rare low grade patient-derived cell lines had the ability to migrate in a 2D and 3D environment and confirm migration in the two pediatric HGG cell lines as previously described (13), since cell migration is a prerequisite for tumor cell invasion and tumor recurrence. Under FCS-supported growth conditions we noted that all cell lines had distinct cell morphologies in *in vitro* cultures highlighting inter-tumoral heterogeneity among these tumors (Figure 2A). Our random 2D migration study revealed that all cell lines possessed distinct migratory abilities (Figures 2B–D). In 2D random migration velocity values ranged from 0.06 to 0.12 μm/min (pLGG cell lines) and 0.1 and 0.24 μm/min (KNS42 and SF188). We also noted differences in the ability to travel with directional persistence (distance traveled over time). We observed values for distance traveled within 72 h of 23.6, 50.7, 61.4, 63.7, and 118.7 μm for IN1520, IN2688, IN1591, IN2017, and IN2356 and 37.2 and 122.7 μm for KNS42 and SF188 (Figure 2D). Interestingly, the velocity values obtained for the pediatric LGG cell lines were similar within this group and approaching the velocity observed for the high-grade cell line KNS42. Directionality varied more among the pediatric LGGs with values on a whole greater than those obtained for KNS42. Among the pLGGs IN2356 exhibited the greatest migratory activity, which was the cell line that did not have the BRAF mutation.

**Figure 3:**
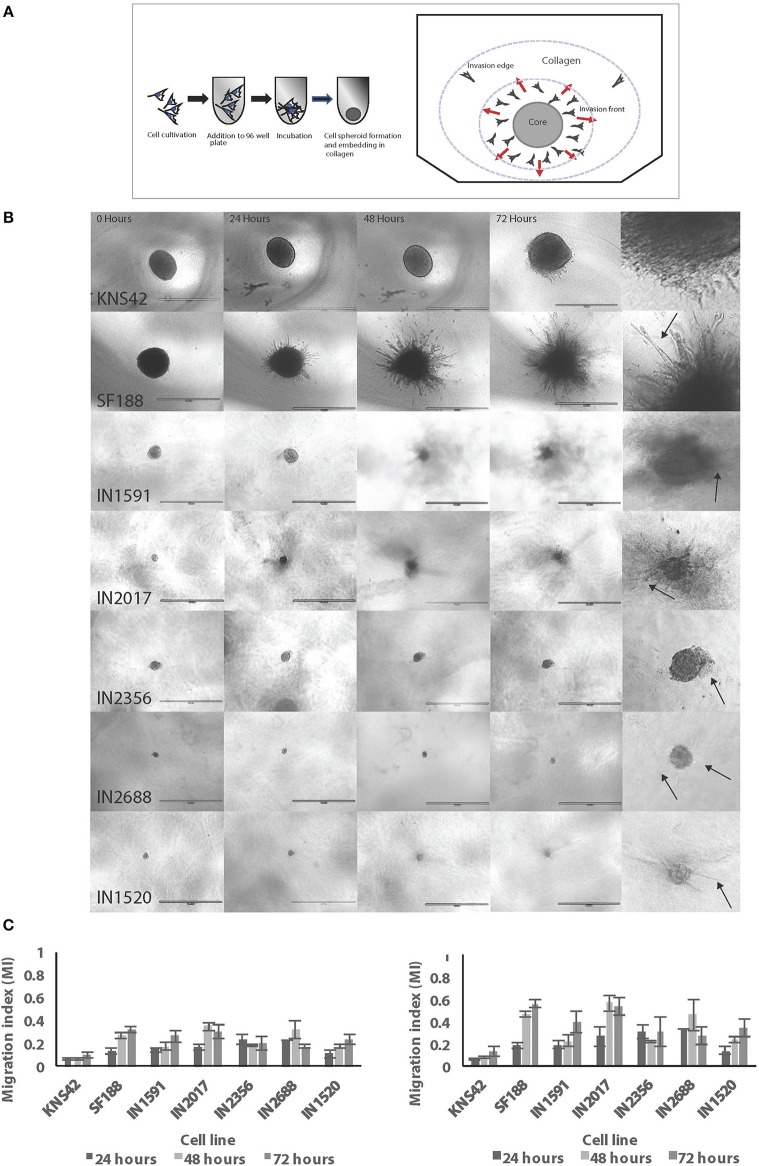
Low and HGGs are able to migrate/invade in a 3D environment as assessed by spheroid migration/invasion assays in collagen. **(A)** Diagram illustrating the measuring zones for establishing migration in 3D. Two zones are measured, the invasion front and invasion edge. Unfilled cell cartoons indicate single cells with potentially increased migratory activity supporting advanced migration/invasion into the collagen as previously observed. **(B)** Representative EVOS brightfield microscopy images for spheroids and migratory cells of the individual cell lines over 72 h. After 72 h all cell lines had migrated/invaded into the collagen. Also shown is a panel of enlarged images for the same cell lines to highlight morphological features of cell migration/invasion of the cell lines in 3D environments. Arrows indicate migrating/invading cells. Scale bar= 1,000 microns. **(C)** Migration/invasion in 3D was measured and expressed as migration indexes (MI) for both the invasion front and edge. Graphs illustrate results at three different time points over 72 h.

We were able to generate spheroids for 3D migration assays for all cell lines investigated (Figures 3A–C). We firstly noted that the generated pLGG spheroids were much smaller than the pHGG spheroids which is attributable to low proliferative rates in the pLGGs, also observed in routine tissue culturing. Interestingly, in the 3D migration assays all LGGs migrated/invaded into the collagen matrix as efficiently as the HGGs, with all migrating at a greater rate than KNS42 (Figures 3B,C). The MI indexes for the migration front were 0.17, 0.2, 0.24, 0.26, and 0.3 for IN2688, IN2356, IN1520, IN1591, and IN2017 and 0.09 and 0.32 for KNS42 and SF188. Migration indexes for the migration edge of 0.28, 0.31, 0.35, 0.4, and 0.54 were obtained for IN2688, IN2356, IN1520, IN1591, and IN2017 and 0.13 and 0.56 for KNS42 and SF188 (Figure 3C). Slight morphological changes over time were observed for the cells migrating away from the original core. The migrating cells in IN1591 emanated from the core in a sheet-like manner. A similar pattern was observed in IN2688 but with more rounded cells. IN2017 consisted of tiny chains and streams of cells extending from its core, suggesting a mixture of chain and stream migration. IN2356 had appendages and streams emanating from the core. In IN1520, there were single and rounded cells detached from the core which could indicate prominent single cell migration rather than clustered migration in addition to string-like protrusions (Figure 3B). As previously described SF188 migrated in a spike like manner with many cells arranged in chains, whereas KNS42 produced cell sheets expanding into the surrounding collagen (13) (Figure 3B).

### Pediatric LGG and HGG Exhibit Varying Levels of FGFR1 and Phosphorylated FGFR1 After Stimulation With FGF2

**Figure 4:**
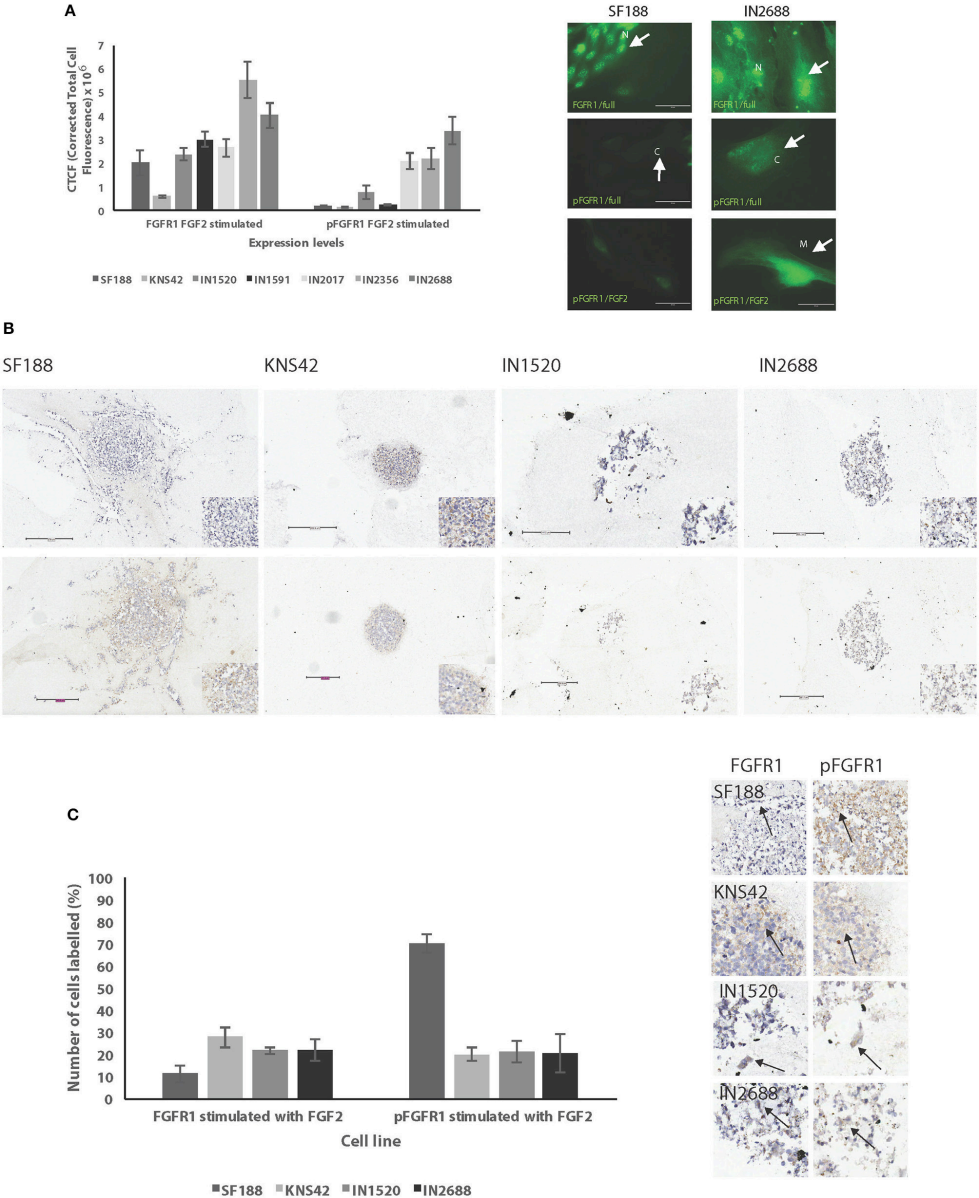
Low and high grade pediatric gliomas exhibit varying levels of FGFR1 and pFGR1. **(A)** Cell lines were assessed for FGFR1 and pFGFR1 levels by immunofluorescence intensity values after stimulation with FGF2. Low grade gliomas exhibited higher levels of both FGFR1 and pFGFR1 than high grade gliomas. Nuclear, cytoplasmic, and membranous localization was observed for pFGFR1 in the cell lines. Green—FGFR1 or pFGFR1 antibody; scale bar= 50 microns. SF188 is shown as a representative example of protein localization in HGGs and IN2688 as a representative example for LGGs. Only FGFR1 or pFGFR1 fluorescence staining patterns are shown to highlight the location of the proteins within the cells. Arrows indicate protein localizations in the cell lines SF188 and IN2688 as representative examples. Note that the camera settings were the same for the cell lines within unstimulated and stimulated groups to allow comparison of protein levels, which is illustrated in the differences in fluorescence levels of SF188 and IN2688 depicted here. **(B)** In 3D spheroids FGFR1 and pFGFR1 levels were also detected in the cytoplasm and membranous locations. Top: FGFR1 expression and localization after stimulation with FGF2. Bottom: pFGFR1 expression after stimulation with FGF2. **(C)** Graphic representation of protein levels in spheroids is shown. The spheroids had been stimulated with FGF2 and were fixed after 72 h. The panel of images shows FGFR1 and pFGFR1 expression after stimulation with FGF2 in SF188, KNS42, IN1520, and IN2688 with membranous and cytoplasmic localization as indicated by the arrows (x 20 magnification); scale bars 100–300 microns.

We went on to investigate the relationship of migratory activity and FGFR1 expression and activity, given that FGFR1 mutations have been recently identified in pediatric gliomas (5) and we had also determined associations of FGFR1 levels and pFGFR1 localization and various clinicopathologic parameters in our TMAs. We first carried out immunofluorescence assays on all cell lines to determine FGFR1 and pFGFR1 levels. We chose this methodology over Western blotting as it proved difficult to obtain the protein levels needed for SDS-PAGE electrophoresis from cell extracts. Serum-free medium supplemented with FGF2 was chosen to determine cell response and protein localization to stimulation with ligand to assess cellular response to stimulation with the preferred ligand FGF2. There were varying levels of FGFR1 and pFGFR1 and protein localization after stimulation with FGF2 between cell lines. We demonstrated the highest FGFR1 levels in IN2356, followed by IN2688, IN1591, IN2017, and IN1520. SF188 had higher FGFR1 levels than KNS42. Interestingly, the highly migratory cell line IN2356 had the highest levels of FGFR1 and was also the only cell line with no BRAF mutation. A similar observation was made for the levels of pFGFR1 for the pediatric LGG; protein levels in SF188 and KNS42 did not differ but were significantly lower than the protein levels observed in the pediatric LGG. We also observed distinct protein localizations: nuclear and cytoplasmic for total FGFR1 and nuclear, cytoplasmic and membrane associated for phosphorylated FGFR1 (Figure 4A, Supplemental Figure 1).

We also analyzed FGFR1 and pFGFR1 levels in spheroids prepared for immunohistochemistry (IHC) derived from IN1520 and IN2688 as well as SF188 and KNS42, which were the cell lines that had generated the largest spheroids we were able to process for IHC. We were able to demonstrate specific FGFR1 and pFGFR1 staining in the spheroids; in spheroids grown in medium supplemented with serum (FCS) all cell lines expressed FGFR1; we only detected pFGFR1 in one cell line, SF188. FGFR1 was localized in the cytoplasm, whereas we noted both cytoplasmic and membranous labeling in the pFGFR1 stain (data not shown). In spheroids grown in FGF2 supplemented-serum free medium, FGFR1 was again detected in all cell lines; this time, pFGFR1 was present in all cell lines examined (Figures 4B,C) indicating FGFR1 activation after stimulation with ligand.

### Cell Migration Is Promoted by FGFR1 Stimulation With FGF2 in Some Pediatric Glioma Cell Lines and Affects Anti-migratory Activity of FGFR1 Inhibitors

**Figure 5:**
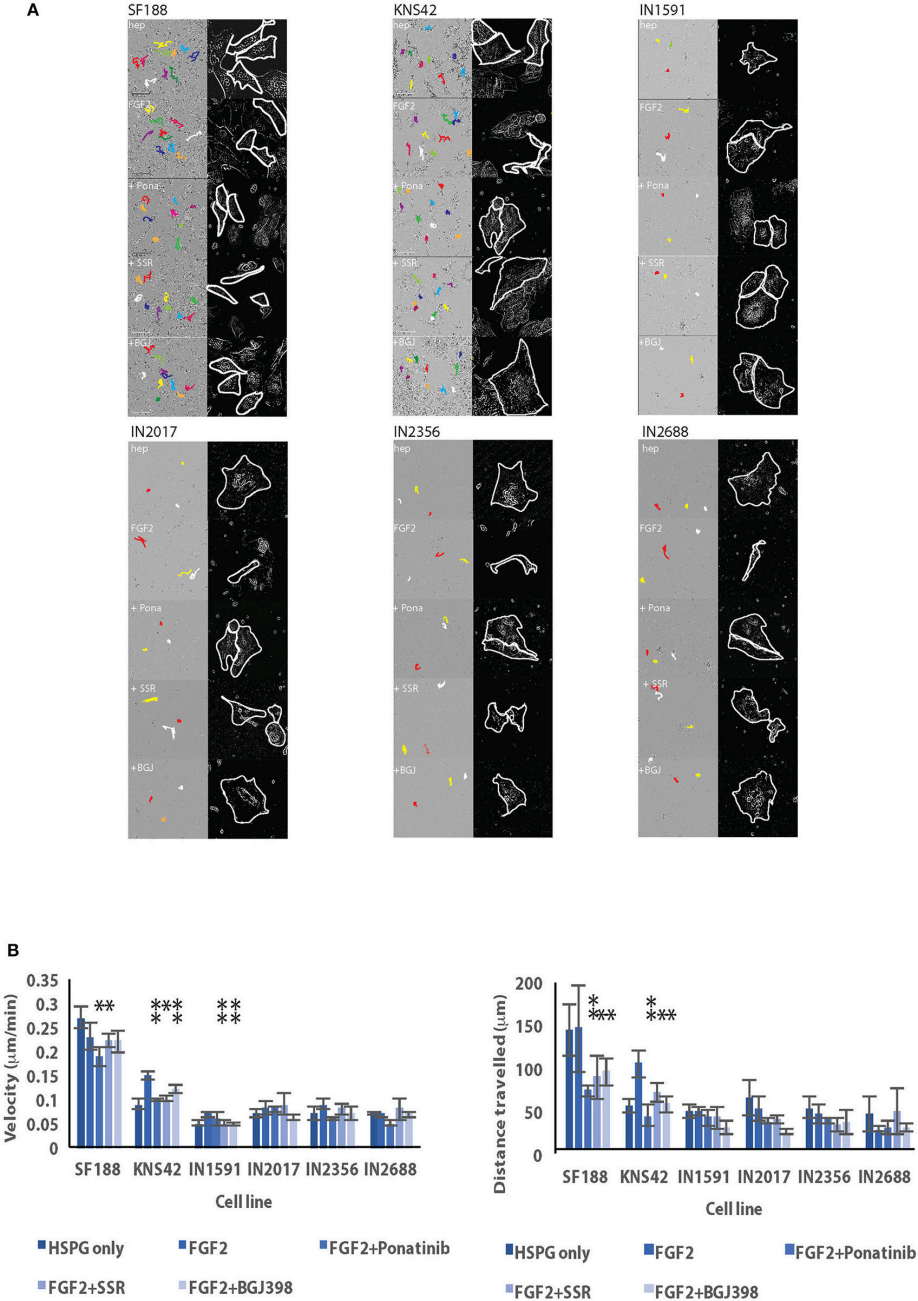
Cell migration is stimulated in serum free conditions after the addition of FGF2 and migratory activity is inhibited in some cell lines after addition of FGFR1 inhibitors in 2D random cell migration. **(A)** Stills of live cell imaging of various low and high grade pediatric cell lines after 72 h reveal anti-migratory effects of inhibitors. Colored lines indicate migration tracts of individual cells. Inset images show traces of individual representative cells for enhanced visualization of cell morphology. All images original magnification x 20. **(B)** Graphic representation of migration in 2D under serum-free conditions after the addition of FGF2 by pLGGs and HGGs. Results for both velocity and directionally persistent migration as indicated by distance traveled are shown. ^*^denotes *p* < 0.05; ^**^p < 0.01.

To assess a potential role for FGFR1 signaling in cell migration we replaced whole medium containing FCS with serum free medium supplemented with either HSPG only or with FGF2/HSPG as ligand for FGFR1 signaling. FGFR1 stimulation with FGF2 under serum-free conditions promoted migration in the pLGG cell lines IN1591, IN2017, and IN2356 and in the pHGG cell line KNS42 and in terms of directionality in KNS42 in 2D (Figures 5A,B) as assessed against cells stimulated with HSPG (hep) only. Phenotypically we observed dramatic changes in cell morphology in some of the cell lines, especially KNS42 where large, flattened cells became elongated and thinner and in the pediatric LGGs IN2017, IN2356 and IN2688 where cells with a similar cell morphology to KNS42 adopted a mesenchymal phenotype with a pronounced cell front and longer cell protrusions in response to stimulation with FGF2 in keeping with a more migratory phenotype (Figure 5A, Supplemental Figure 2).

We next asked if FGFR1 driven migration could be inhibited by the activity of three different FGFR1 inhibitors differing in their specificity against FGFRs. We observed anti-migratory effects with the inhibitors Ponatinib (Pona), BGJ398 (BGJ), and SSR128129E (SSR) at pre-determined anti-migratory concentrations. In 2D random cell migration, SF188 velocity was affected by Ponatinib and SSR128129E and all three inhibitors reduced cell velocity in KNS42. IN1591 was affected by treatment with SSR128129E and BGJ398. Directionality was reduced by all three inhibitors in only the two high grade gliomas, SF188 and KNS42 (Figures 5A,B).

**Figure 6:**
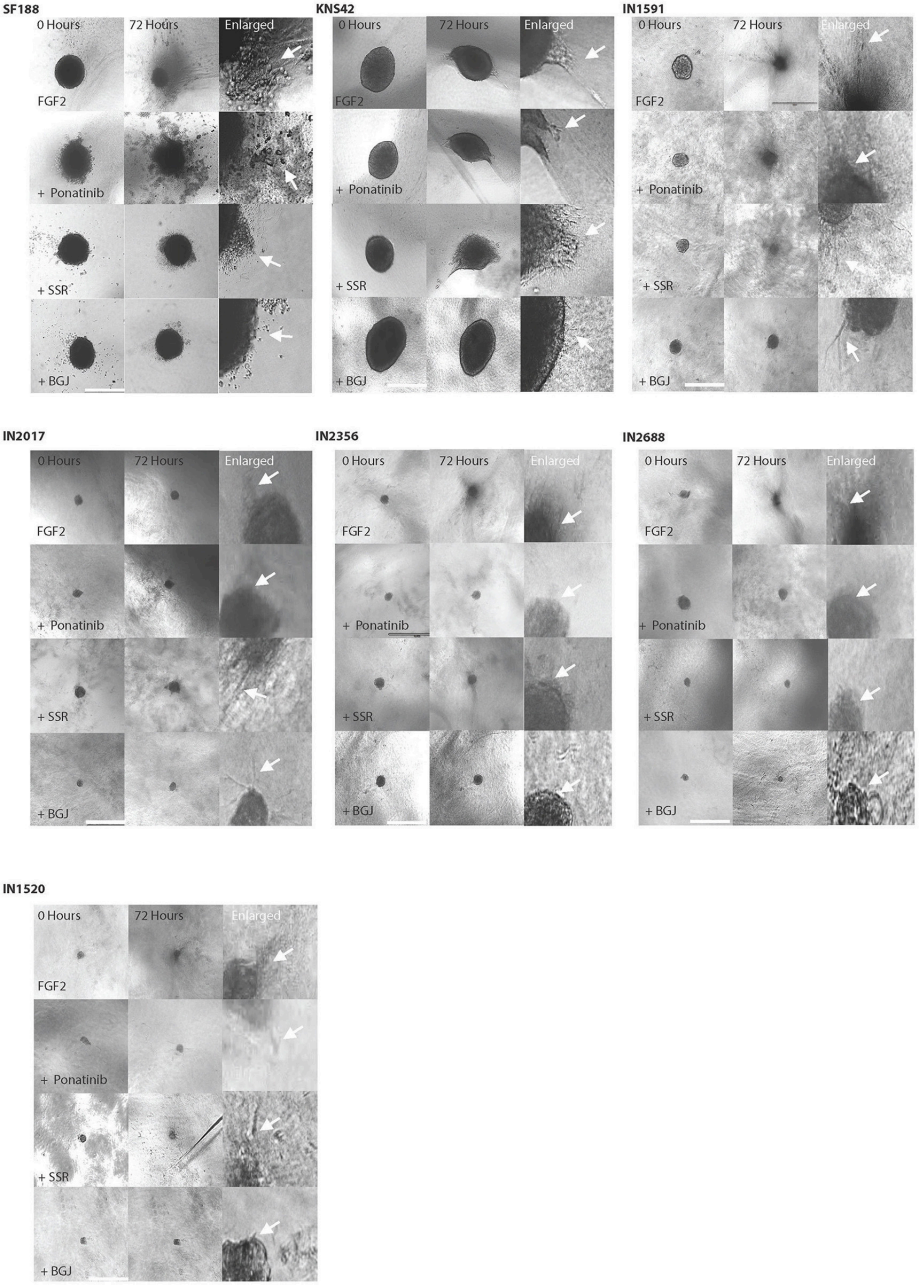
Cell migration is stimulated in serum free conditions after the addition of FGF2 and migratory activity is inhibited in some cell lines after addition of FGFR1 inhibitors in 3D invasion assays. Representative images of spheroids allowed to migrate under serum free conditions and with the addition of FGF2 indicate the inhibitory activity of various FGFR1 inhibitors after treatment with inhibitors (Ponatinib, SSR and BGJ). Examples are shown for the HGGs and LGGs at beginning of the experiment and after 72 h (x 40). Also shown are images of the spheroid at higher magnification to allow visualization of morphological features of migratory cells close to the spheroid edge (labeled enlarged) as indicated by the arrows.

**Figure 7:**
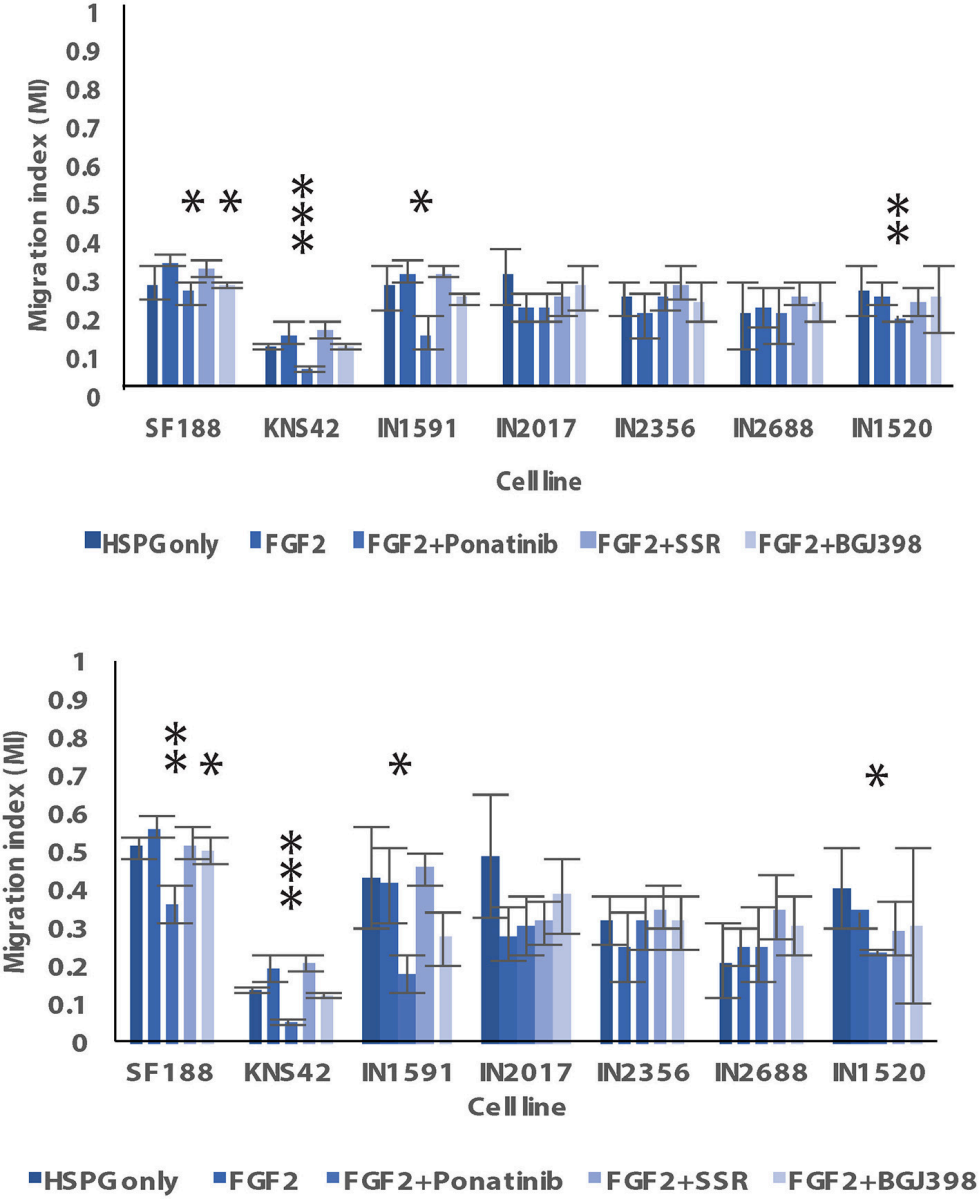
Graphic representation of migration by pLGGs and HGGs in 3D under serum-free conditions after the addition of FGF2. As a control, spheroid migration under serum free conditions with HSPG only are also shown. FGF2-stimulation was in combination with HSPG. Results are shown for both the effect of the inhibitors on the migration front **(Top)** and edge **(Bottom)**. ^*^Denotes ^*^p < 0.05; ^**^p < 0.01; ^***^p < 0.001.

In the 3D migration assays, we also observed morphological changes in the migratory cells, especially in KNS42, where we observed elongated protrusions and in SF188 rounded individual cells and in IN1591 spheroids with more pronounced string-like protrusions and IN2688 with more prominent cell sheets (Figure 6, Supplemental Figure 3). Overall, there were more noticeable effects on cell migration of the migration front than the migration edge after stimulation with FGF2 under serum-free conditions; FGF2 induced enhanced migration in SF188 and KNS42 and pLGG IN1591 and IN2688, which were the cell lines with clear morphological changes (Figure 6, Supplemental Figure 3). In the migration edge only the pHGG cell lines were affected by FGFR1 stimulation as indicated by an increase in MI values (Figure 7). Reduced migration front indexes were recorded after addition of Ponatinib (KNS42) and Ponatinib and BGJ398 (SF188), and in IN1591 and IN1520 with Ponatinib only. Interestingly, in two low grade cell lines, IN2017 and IN2356, there was a trend of inhibitors to enhance cell migration (Figure 7). For the migration edge, decreased migration was observed in SF188 with BGJ398 and in KNS42 with Ponatinib, as well as in IN1591 and IN1520 with Ponatinib. As for the migration front there were trends in IN2017, IN2356, and IN2688 for increased cell migration after treatment with the three inhibitors (Figure 7). Markers for apoptosis (cleaved caspase 3) remained unchanged in the spheroids tested, confirming that the observed inhibitor effect resulted from anti-migratory activity and not cytotoxicity; proliferation (Ki67) was affected after treatment with SSR128129E in IN1520, KNS42, and SF188 and in KNS42 after treatment with BGJ398 (Tables 6, 7).

**Table 6:** Ki67 expression in FGF2-stimulated spheroids without and with inhibitor treatment.

	**IN2688**	**IN1520**	**KNS42**	**SF188**
Ki67 FGFR1 stim	Low	High	High	High
Ki67 FGFR1 stim +Ponatinib	Low	High	High	High
Ki67 FGFR1 stim + SSR	Low	Low	Low	Low
Ki67 FGFR1 stim + BGJ	Low	High	Low	High

**Table 7:** Cleaved caspase 3 expression in FGF2-stimulated spheroids without and with inhibitor treatment.

	**IN2688**	**IN1520**	**KNS42**	**SF188**
CC3 FGFR1 stim	Low	Low	Low	High
CC3 FGFR1 stim +Ponatinib	Low	Low	Low	High
CC3 FGFR1 stim + SSR	Low	Low	Low	High
CC3 FGFR1 stim + BGJ	Low	Low	Low	High

## Discussion

Brain tumors are the most common solid tumors of childhood with high mortality rates highlighting the clinical importance of exploring new therapeutic targets to combat this devastating disease (18, 19). Identification of potential targets must take into account the need for combination treatments to avoid resistance to inhibition of specific kinase signaling pathways and one of these potential targets is FGFR1. Following recent reports of recurrent FGFR1 mutations in pediatric gliomas and known roles for FGFR1 in invasion in other cancer types, we firstly wanted to determine clinical relevance of FGFR1 expression. Our TMA results revealed increased FGFR1 expression in malignant tumors in comparison to benign tumors. We also recorded an overall finding that associated high FGFR1 levels with low grade tumors suggesting a role of FGFR1 in tumorigenesis. We also found evidence of an association of activated FGFR1 when localized in the cell membrane with malignancy and tumor grade (2 and 3). This may be indicative of a role of activated FGFR1 and tumor progression. In the subset of pediatric and young adult samples in this cohort we found a negative association of FGFR1 expression and age; based on a small sample size these results must be validated by establishment of large scale TMAs. We next investigated the potential for cell migration in low and high grade pediatric gliomas as a prerequisite for disease progression and recurrence and, next, assessed the role of FGFR1 in cell migration and invasion for chemotherapeutic intervention (20–23). This is a novel therapeutic approach, targeting a hallmark feature of brain tumor pathogenesis and has to our knowledge not been investigated in pediatric gliomas beyond genomic analyses. We describe for the first time the migratory behaviors of rare low grade pediatric patient cell lines. All cell lines displayed a specific migratory phenotype and this was largely independent of glioma grade. This demonstrates differences in migratory behaviors amongst tumors of the same grade, highlighting that each tumor is unique with specific cell migration and invasion patterns. Targeting cell migration and invasion with treatment should reflect this uniqueness particularly when considering combination treatment with inhibitors targeting other signaling pathways.

Both pediatric LGG and HGGs possessed varying levels of FGFR1 and pFGFR1 in 2D monolayers and cells maintained within 3D spheroids. The ability of pediatric LGGs to form spheroids in *in vitro* systems is a novel observation and indicates the potential of using this model for further studies on tumorigenesis and applicability in drug screens. Interestingly, in pediatric LGGs, FGFR1 levels were higher than in HGGs in 2D monolayers but in 3D environments activated FGFR1 levels were higher in the highly migratory SF188 suggesting differential roles of FGFR1 signaling among low and high grade gliomas depending on the microenvironment. In FGFR-FGF2 stimulation assays we also determined that some of the cell lines responded to stimulation with morphological changes from a rounded to a more elongated mesenchymal phenotype and potentially enhanced migration. There was an increase in velocity and reduction in directionality in the cell lines apart from IN2688. Reduced directionality in the cell lines may indicate a loss of cell polarity following FGFR1 stimulation (24, 25). Previous studies have demonstrated FGFR1 activation caused a loss of cell polarity in breast cancers (26). In 3D, the pediatric HGG both had increased migration indexes in HSPG/FGF2 compared to HSPG only controls which suggested pro-migratory effects of FGFR1 mirroring the observed protein expression levels of activated FGFR1. In contrast, the pediatric LGG were mixed in their responses to FGFR1 stimulation with increased or decreased migration as well as morphological changes observed in the spheroids with increased numbers of migrating cells highlighting the heterogeneity among low grade tumors.

Treatment with Ponatinib, a multi-targeted tyrosine kinase inhibitor, BGJ398, a pan-FGFR inhibitor with VEGFR activity, and SSR128129E, a highly specific FGFR1 inhibitor induced different responses in the cell lines (27–29). The greatest overall reduction seen across all inhibitors was with BGJ398, which suggests one of its other targets (i.e., VEGFR2 and/or FGFR2-4) may contribute to cell migration as well as FGFR1 indicating the involvement of other kinase signaling. We propose to include BGJ398 in future *in vitro* treatment combination studies targeting BRAF in patients with BRAF mutations to investigate potential new therapeutic avenues. We also suggest further testing of BGJ398 in combination and as stand-alone in BRAF wildtype/FGFR1 mutation patients.

Differences were seen in FGFR1 expression at the protein level between pediatric LGG and HGG. Differences in expression between tumor grades have been well established in adult gliomas (30). However, our immunofluorescence results indicated higher FGFR1 expression in pediatric LGG than HGG, which is opposite to the observations in adult gliomas supporting pediatric gliomas to be biologically distinct from adult gliomas (31).

We demonstrate migratory phenotypes of low grade pediatric astrocytoma cells in a 2D and 3D environment, varying expression of FGFR1 and its activated form at the protein level and finally distinct responses to FGFR1 inhibition. We also observed varying anti-migratory effects with the inhibitors used on pediatric HGG. Reports from other cancer types targeted with FGFR1 inhibitors support the notion that inhibitors do not have equivalent efficiency varying from one tumor type and drug family to another suggesting the *FGFR* alterations have different biological meanings (32). Inhibitor effects appear to be cell line specific, highlighting the need for personalized treatment of pediatric gliomas. Although our study did not reveal mutations in the reported hotspots additional published mutations should be analyzed in our sample set and we also hypothesize that FGFR1 overexpression alone can drive tumor progression in pediatric gliomas, which needs to be explored in further studies.

There is compelling evidence that FGFR1 signaling has a significant role in glioma tumorigenesis and progression. FGFR1 expression is positively associated with more invasive and malignant adult gliomas and thus positively associated with poorer prognoses (30). Given the lack of treatment options for relapsed and refractory pediatric HGGs, future work should focus on investigating FGFR1 signaling as a chemotherapeutic target for this patient group (31, 32). There is scope that targeting FGFR1 signaling on its own or in combination has the potential to improve prognosis. Future studies must address the role of FGFR1 in pediatric gliomas and its effect on the different tumor hallmarks is needed (33, 34).

## Availability of Data and Material

The data and material supporting the study are stored in the Leeds Institute of Cancer and Pathology, University of Leeds.

## Author Contributions

NE and AB-R conceived and carried out experiments. JC, MH, AI, FE, RM, CT, and TW carried out experiments. NE and AB-R conceived experiments and analyzed data. SS critically reviewed the manuscript. All authors were involved in writing the paper and had final approval of the submitted and published versions.

### Conflict of Interest Statement

The authors declare that the research was conducted in the absence of any commercial or financial relationships that could be construed as a potential conflict of interest.

## Footnotes

^1^(https://imagej.nih.gov/ij/)

## Supplementary Material

Supplemental Figure 1 Immunofluorescence images for SF188 and IN2688 labeled for FGFR1 (green), pFGFR1 (green), actin (phalloidin, red), and DNA (DAPI, blue) and merged images of the three channels. The panels are presented in the same order as shown for the images in Figure 4. Scale bar = 50 microns.

Supplemental Figure 2 Graphic representation of morphological features observed in 2D cultures in response to stimulation with FGF2 ligand and treatment with inhibitor. Morphological changes in cell size were observed after stimulation with FGF2 and treatment with inhibitor as indicated.

Supplemental Figure 3 Graphic representation of morphological features observed in migratory cells of 3D spheroid cultures in response to stimulation with FGF2 ligand and treatment with inhibitor. Morphological changes in migrating cells away from original spheroid cores were observed after stimulation with FGF2 and treatment with inhibitor.
