# Supplementary figures and images for: FGFR1 Expression and Role in Migration in Low and High Grade Pediatric Gliomas
Source: Front Oncol. 2019 Mar 13;9:103. doi: 10.3389/fonc.2019.00103 (PMC6425865; doi:10.3389/fonc.2019.00103)

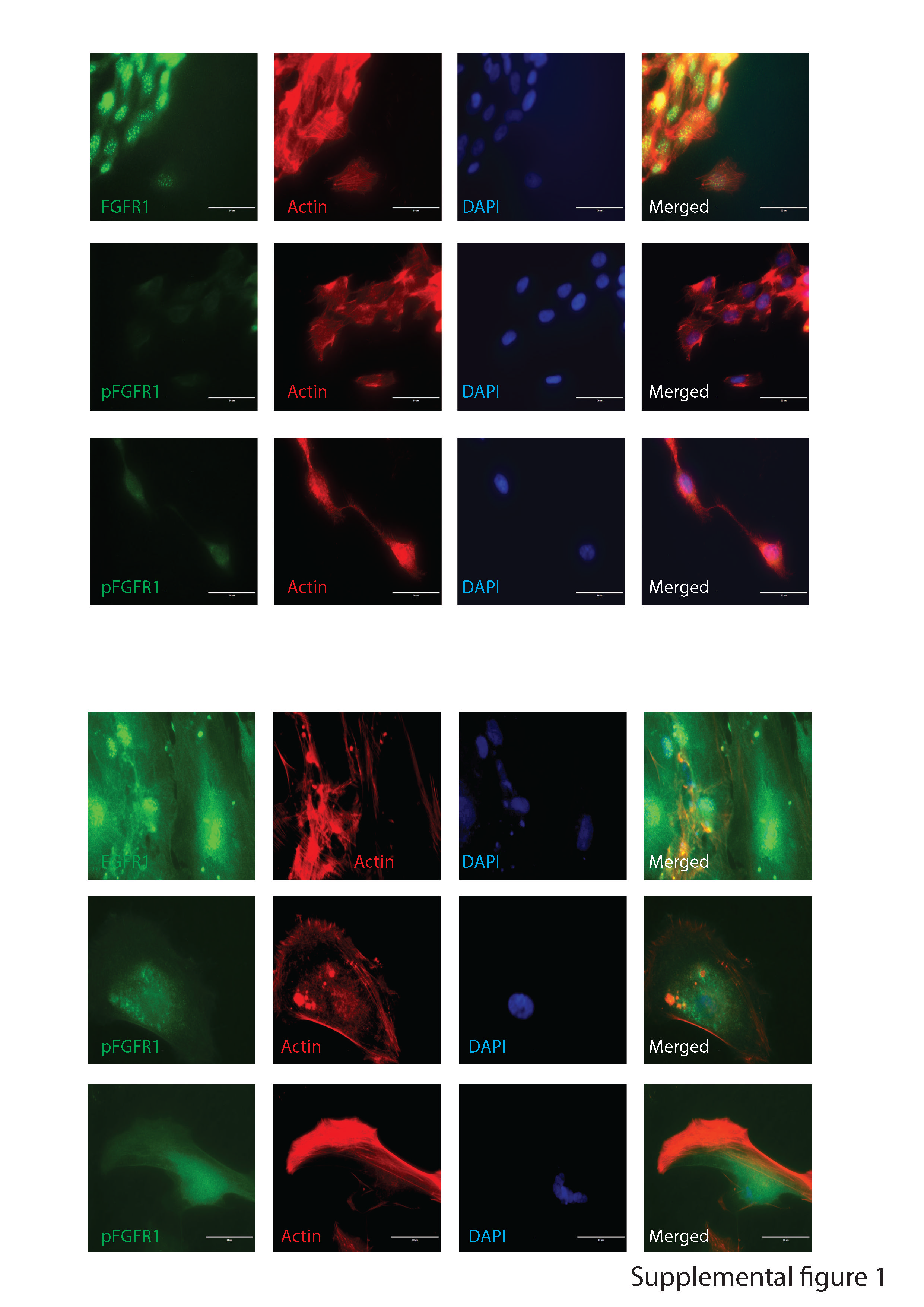

Supplement: Supplemental Figure 1 — Immunofluorescence images for SF188 and IN2688 labeled for FGFR1 (green), pFGFR1 (green), actin (phalloidin, red), and DNA (DAPI, blue) and merged images of the three channels. The panels are presented in the same order as shown for the images in Figure 4. Scale bar = 50 microns. [file Image_1.TIFF]
